# Is the biology of breast cancer changing? A study of hormone receptor status 1984–1986 and 1996–1997

**DOI:** 10.1038/sj.bjc.6604934

**Published:** 2009-02-17

**Authors:** S B F Brown, E A Mallon, J Edwards, F M Campbell, L M McGlynn, B Elsberger, T G Cooke

**Affiliations:** 1 Department of Surgery, Crosshouse Hospital, Glasgow Royal Infirmary, Alexandra Parade, Glasgow G31 2ER, Scotland, UK; 2 Department of Pathology, Western Infirmary, Dumbarton Road, Glasgow G116NT, Scotland, UK

**Keywords:** breast, epidemiology, hormone receptor status, survival

## Abstract

Using archived tumours, those from 1984–1986 and 1996–1997 underwent immunohistochemistry for hormone receptors and grade analysis. A significant shift towards more ER-positive and low-grade disease was found; this appears to reflect screening practices, but could still influence survival.

Breast cancer is the commonest cancer in women in the United Kingdom, with over 40 000 cases diagnosed annually, and the incidence is increasing. Survival rates are also increasing partly due to advancements in hormonal and chemotherapeutic management, in addition to a trend towards multidisciplinary management and specialist surgeons. The UK nationwide mammographic screening programme was also designed to reduce mortality. It has been suggested that the epidemiology of breast cancer may have changed over time, with more oestrogen receptor (ER)-positive tumour than in the past (Pujol *et al*, 1994; Bradburn *et al*, 1998; Li *et al*, 2003; Glass *et al*, 2007). However, studying retrospective data on ER status has the disadvantage that the assays used to establish ER status, and hence their sensitivity and specificity have changed over time. We, therefore, examined trends in the molecular biology of breast cancers in patients from two large centres in Glasgow by performing immunohistochemistry on archived tumour samples, thereby avoiding artefactual changes in receptor status over time. We also examined the survival of study patients.

## Materials and methods

### Patient selection

This study aimed to compare the molecular phenotype of stored tissue samples from two separate cohorts of patients, defined by the period in which they had their surgery. All female patients who had surgery for operable breast cancer at two teaching units in Glasgow during 1984–1986 and 1996–1997 were identified. The study had the approval from local ethics committee. Full pathological, demographic, screening and 5-year survival data were either available from the Scottish Cancer Registry or obtained from the patient's case record or pathology records. Deprivation status was ascertained using established postcode Carstairs deprivation categories (1–7) derived from 1981 or 1991 census data. For each patient, an archived paraffin-embedded tumour block was searched for within the relevant pathology department. Following sample size determination, there were originally 1076 patients (423 in 1984–1986 (cohort 1) and 653 in 1996–1997 (cohort 2)) from which 900 tumour blocks were available for analysis (323 and 577 in cohorts 1 and 2, respectively).

Tumour sections were prepared according to routine pathological techniques, and then sent to a pathologist for determination of tumour grade using the modified Scarff–Bloom–Richardson scale and marking of suitable tumour areas. Three 0.6-mm circular cores were then taken from the marked areas in each tumour block and placed into paraffin blocks in tissue microarray format. Sections from each block were taken for ER immunohistochemistry, Her-2 receptor and progesterone receptor (PR) to be performed; each full set of sections underwent ER, PR or HER-2 immunohistochemistry at the same time. In all, 862 of the 900 samples (95%) underwent grade analysis, and 20% of the tumours in cohort 1 and 19% of tumours in cohort 2 did not undergo ER immunohistochemistry due to fragmented cores or absence of tumour in the core. For the same reasons, 14% of tumours in cohort 1 and 10% in cohort 2 did not undergo PR immunohistochemistry, and 15% of tumours in cohort 1 and 18% of tumours in cohort 2 did not undergo Her-2 staining.

Oestrogen receptor immunohistochemistry was carried out using Novocastra 6F11 mouse antihuman ER (Novocastra, Newcastle-Upon-Tyne, UK) with a manual protocol at a dilution of 1 : 50, with epitope retrieval carried out using EDTA at pH 8.0 with a microwave pressure cooker technique for 5 min. After the primary antibody step, slides were refrigerated overnight, with the rest of the steps carried out at room temperature. Progesterone receptor immunohistochemistry was carried out using Dako 636 mouse antihuman PR (Dako, Ely, UK), using a dilution of 1 : 50 and epitope retrieval using citrate pH 6.0 and a microwave pressure cooker technique for 5 min, with the final protocol being carried out at room temperature using a Dako Autostainer. Her-2 immunohistochemistry was carried out in a Dako Autostainer at room temperature using the standard Dako Herceptest protocol.

### Analysis

Once immunohistochemistry had been carried out, each core was assessed by light microscopy and scored by an experienced scorer using a weighted histoscore ((% tumour cells scoring at intensity 1)+(2 × % scoring at intensity 2)+(3 × % scoring at intensity 3)). As each tumour had been cored in triplicate, a mean histoscore for each core was calculated. For ER and PR, ‘positive’ was taken as a histoscore of 10 or over, and for Her-2, positivity was taken as a histoscore of 90 or over. A second experienced scorer examined 10% of the cores (Kirkegaard *et al*, 2006). Statistical analysis was performed using SPSS statistics software (SPSS, Chicago, IL, USA), version 14.0. Comparison of the demographics of the two groups was carried out using *χ*^2^-analysis (Fisher's exact test in one case where numbers were small). Comparison of the molecular profiles in the groups was carried out using *χ*^2^-analysis for hormone receptor or grade status, *t*-test to compare mean receptor levels and Mann–Whitney test to compare median receptor levels. A multivariate analysis was performed using binary logistic regression to assess whether age, screening and deprivation affected the percentage of ER-positive tumours in the groups. Initial tests suggested that there was not a direct linear relationship between ER and age, but ER-positivity rates increased from 60 years of age, and hence ‘age over or under 60’ as a categorical variable was included in the regression. Kaplan–Meier survival analysis (censored to 5 years) was carried out, using the log-rank test to test for difference between the groups. Stepwise Cox's proportional hazard regression was carried out to compare factors influencing survival.

## Results

The demographics of the patients whose tumour blocks were available are seen in Table 1. All tumours in cohort 1 had been detected symptomatically rather than by screening, the screening programme having yet to be introduced in Scotland.

A comparison of the results of immunohistochemistry based on cohort (and screening status within the 1996–1997 cohort) is presented in Table 2. In a multivariate analysis, the significance of the impact of cohort period (1984–1986 or 1996–1997) on ER status did not persist after correction for percentage of patients aged over 60 and screening status, in combination with other factors or on their own. Oestrogen receptor positivity within each 10-year-age range increased from 1984–1986 to 1996–1997 (except in those under 30 years), although the numbers involved meant that this did not approach statistical significance. Table 3 shows Cox's proportional hazard regression analysis of survival; when the effect of the period of diagnosis on survival was adjusted for differences in ER status of the patients alone, the period of diagnosis (i.e., 1984–1986 or 1996–1997) remained as a significant independent factor in survival. After correcting, for all the factors in the model, the effect of the period of diagnosis on survival persisted, with survival being higher in 1996–1997.

## Discussion

A significant change in grade distribution over time was seen in this study, particularly a reduction in the frequency of grade 3 and an increase in the frequency of grade 1 tumours, a change that appeared to be due to screen-detected tumours in cohort 2. The pathological grade of screen-detected tumours has received much attention in the literature, these being of lower grade than symptomatically detected tumours; it is uncertain, however, whether this represents an interruption of ‘phenotypic drift’ or simply a longer asymptomatic preclinical phase (Tabar *et al*, 1999).

This study also showed an increase over time in the percentage of breast cancers that were ER positive. The increase from 64.2 to 71.5% was significant on *χ*^2^-analysis. The increase did not persist on logistic regression after adjusting for the prevalence of patients over 60 in the groups. However, an increase in the percentage of ER positivity within each 10-year-age group (except those under 30 years) in the study period, although not reaching statistical significance because of the numbers in each subgroup, suggests a trend towards the overall more ER-positive disease in cohort 2. The ER-positive rise also did not persist after adjustment for the screening status of the patients in the groups. This almost certainly reflects the fact that screen-detected tumours are slower growing, and hence more likely to be ER positive than negative. As there is unlikely to be any phenotypic drift from ER-positive to ER-negative status within breast cancers, it is possible that the screening programme has merely detected an excess of ER-positive breast cancers, which have developed as a result of a true change in biology.

There was a significant change in combined ER/PR receptor status over time, most notably a marked decrease in the percentage of tumours that had the poor prognostic ER-negative/PR-negative status. The percentage of tumours that were PR positive and Her-2 positive did not change over time; notably, there was no change in mean score over time for any of the three receptors.

A study of incidence rates of ER-negative and ER-positive breast cancers in a US health plan found that the incidence rate for ER-negative disease had remained relatively constant with a decline from 1999 onwards, whereas for ER-positive disease, there was a significant increase in incidence throughout the study period (Glass *et al*, 2007). Other studies have also suggested an increase in percentage of ER positivity over time (Pujol *et al*, 1994; Bradburn *et al*, 1998; Li *et al*, 2003). In most of the studies of trends in ER status over time, the assays and criteria used to determine ER positivity changed several times during the study periods. Critically, in this study, we used immunohistochemistry on all samples, thereby ruling out an artefactual increase. Furthermore, all samples underwent immunohistochemistry together to eliminate the potential effect of changing laboratory conditions on staining. The study was powered to detect a 10% difference in ER-positive prevalence, and is hence slightly underpowered to detect the observed 7% difference. The inability to retrieve tumour block for all patients and fragmented samples (factors common to studies involving tissue microarrays) reduced the number of samples in each cohort that were analysed, but the tumours that underwent analysis should be representative of the whole cohort.

One explanation for a preferential increase in ER-positive tumours could be a population-wide change in the prevalence of factors that increase the frequency of these tumours, such as late age at first pregnancy, postmenopausal obesity (Potter *et al*, 1995; Colditz *et al*, 2004) and use of hormone replacement therapy (HRT) (Potter *et al*, 1995). There is evidence that the percentage of all children being born to mothers aged 35 years and over is increasing in Scotland, and that mean BMI and prevalence of obesity are increasing (Brown *et al*, 2007). Data on HRT use by the patients in this study were not available.

A change in ER positivity could influence the survival. In this study, breast cancer-specific survival in the 1984–1986 cohort was significantly lower than in 1996–1997. When the effect of period of diagnosis (i.e., 1984–1986 or 1996–1997) on survival was adjusted for the ER status of the patients alone, the period of diagnosis remained a significant independent factor in survival (with survival being higher in 1996–1997). As expected, the difference in survival between cohorts is not fully explained by differences in ER status; treatment and global management changes have undoubtedly contributed to changes in survival over time (Bradburn *et al*, 1998; Thomson *et al*, 2004). However, a true change in ER status could also have implications for the application of data from clinical trials carried out in previous decades to the women of today, as a change in the prevalence of ER-positive disease could alter the overall survival benefit seen from chemotherapy and different hormonal therapies.

## Figures and Tables

**Table 1:** Patient demographics

	**1984–1986**	**1996–1997**	***P* for difference**
Mean age at diagnosis	56.9	58.4	0.049
Median age at diagnosis	59	58	0.179
Percentage detected at screening	0	29	<0.001
			
*Percentage of patients in each deprivation category*			
Affluent	12	17	
Intermediate	41	47	0.05
Deprived	47	36	
*Node positive*	59.3	42.4	0.001

**Table 2:** Results

	**Cohort 1 1984–1986**	**Cohort 2 1996–1997**	**Cohort 2 Symptomatic**	**Cohort 2 Screened**
Grade distribution: percentage grades 1, 2 and 3	8, 49.2 and 42.9	14.9, 48.3 and 36.8	12.2, 46.8 and 41	22.3, 53 and 24.7
		*P*=0.009 for *vs* cohort 1	*P*=0.2 for *vs* cohort 1	*P*<0.001 for *vs* cohort 2 symptomatic
ER-positive tumours	64.2%	71.5%	68.8%	78.4%
		(*P*=0.042)	*P*=0.325 for *vs* cohort 1	*P*=0.024 for *vs* cohort 2 symptomatic
Mean ER score	97.1	102		
		(*P*=0.454)		
Median ER score	104.2	120		
	(IQR 0–190)	(IQR 0–180)		
		(*P*=0.774)		
PR-positive percentage	44.9	49.9		
		(*P*=0.181)		
Mean PR score	41.2	37.9		
		(*P*=0.418)		
Median PR score	0	8.3		
	(IQR 0–80)	(IQR 0–61)		
		(*P*=0.181)		
Her-2-positive percentage	21.5%	20.6%		
(*P*=0.170)				
Mean Her-2 score	52.2	43.1		
		(*P*=0.772)		
Median Her-2 score	0	0		
	(IQR 0–50)	(IQR 0–67)		
		(*P*=0.773)		
ER+/PR+ percentage	42.4	46.7		
ER+/PR− percentage	21.8	24.8		
ER−/PR− percentage	33.3	23.5		
ER−/PR+ percentage	2.5	5		
		(*P*=0.023)		
	0.620	0.887	0.874	0.976
5-year breast cancer cumulative survival		(*P*<0.001)	*P*<0.001 for *vs* cohort 1	*P*=0.148 for *vs* cohort 2 symptomatic

**Table 3:** Cox's proportional hazard analysis

**Variable**	***P*-value**	**Hazard ratio of death**	**95% confidence interval**
1984–1986 cohort	<0.001	3.43	3.87–4.71
ER-negative status	<0.001	4.29	1.01–1.04
Increasing age (per year)	0.09	1.02	1.01–1.03
Tumour size (per 1 mm rise)	0.01	1.02	−0.63–1.21
Affluent socioeconomic *vs* deprived	0.08	0.29	0.41–1.23
Intermediate socioeconomic *vs* deprived	0.345	0.82	2.18–3.11
Node-positive status	<0.001	2.65	2.99–3.87
Her-2 status in model	Not included in model	Not included	
